# Deletion of Kinin B2 Receptor Alters Muscle Metabolism and Exercise Performance

**DOI:** 10.1371/journal.pone.0134844

**Published:** 2015-08-24

**Authors:** Felipe C. G. Reis, Anderson S. Haro, Aline V. N. Bacurau, Sandro M. Hirabara, Frederick Wasinski, Milene S. Ormanji, José B. N. Moreira, Beatriz H. Kiyomoto, Clelia R. A. Bertoncini, Patricia C. Brum, Rui Curi, Michael Bader, Reury F. P. Bacurau, João B. Pesquero, Ronaldo C. Araújo

**Affiliations:** 1 Department of Biophysics, Universidade Federal de São Paulo, São Paulo, Brazil; 2 School of Physical Education and Sport, University of São Paulo, São Paulo, Brazil; 3 Department of Physiology and Biophysics, Institute of Biomedical Sciences, University of São Paulo, São Paulo, Brazil; 4 CEDEME, Universidade Federal de São Paulo, São Paulo, Brazil; 5 K.G. Jebsen Center of Exercise in Medicine, Norwegian University of Science and Technology (NTNU), Trondheim, Norway; 6 Department of Neurology and Neurosurgery, Universidade Federal de São Paulo, São Paulo, Brazil; 7 Max-Delbrück-Center for Molecular Medicine (MDC), Berlin, Germany; 8 School of Arts, Sciences and Humanities, University of São Paulo, São Paulo, Brazil; Hosptial Infantil Universitario Niño Jesús, CIBEROBN, SPAIN

## Abstract

Metabolic syndrome is a cluster of metabolic risk factors such as obesity, diabetes and cardiovascular diseases. Mitochondria is the main site of ATP production and its dysfunction leads to decreased oxidative phosphorylation, resulting in lipid accumulation and insulin resistance. Our group has demonstrated that kinins can modulate glucose and lipid metabolism as well as skeletal muscle mass. By using B2 receptor knockout mice (B2R^-/-^) we investigated whether kinin action affects weight gain and physical performance of the animals. Our results show that B2R^-/-^ mice are resistant to high fat diet-induced obesity, have higher glucose tolerance as well as increased mitochondrial mass. These features are accompanied by higher energy expenditure and a lower feed efficiency associated with an increase in the proportion of type I fibers and intermediary fibers characterized by higher mitochondrial content and increased expression of genes related to oxidative metabolism. Additionally, the increased percentage of oxidative skeletal muscle fibers and mitochondrial apparatus in B2R^-/-^ mice is coupled with a higher aerobic exercise performance. Taken together, our data give support to the involvement of kinins in skeletal muscle fiber type distribution and muscle metabolism, which ultimately protects against fat-induced obesity and improves aerobic exercise performance.

## Introduction

The kallikrein-kinin system (KKS) is a multi-enzymatic system in which function is mediated by the interaction of peptides called kinins with two different G-protein-coupled receptors, B1 and B2 (B1R and B2R, respectively). Our group presented for the first time the involvement of kinin B1 receptor in metabolism and insulin secretion, using the B1R knockout mice [1, 2]. Additionally, initial evidences regarding a potential involvement of KKS with skeletal muscle (SM) metabolism were reported two decades ago; muscle blood flow and skeletal muscle glucose uptake can be modulated by bradykinin via the constitutively expressed B2 receptor [3–5]. Additionally, absence of B2R resulted in lower tissue sensitivity to insulin and glucose uptake [6]. Later, we demonstrated that B2R^-/-^ mice presented increased postnatal protein body mass, gastrocnemius mass and decreased expression of myostatin in gastrocnemius in postnatal period in comparison to WT counterparts [7].

Total muscle mass highly influences the energy homeostasis [8] and the imbalance between energy intake and energy expenditure are associated to pathologies like obesity and type 2 diabetes. Furthermore, SM fiber type distribution, in addition to total muscle mass, is an important determinant of the profile of energy substrates (i.e. glucose and fatty acids) utilized by SM and physical performance. In this context, mitochondrial content and activity is a distinctive feature among the different SM fiber types [9, 10].

Thus, it is tempting to speculate whether B2R activation could modulate total skeletal muscle mass leading to changes in energy expenditure. Therefore, considering these findings and the fact that the role of B2 receptor in SM has been poorly investigated, the aim of this study was to evaluate the B2R function in skeletal muscle fiber distribution and mitochondrial biogenesis in mice and how this receptor can contribute to an improvement of metabolic responses, diet-induced obesity (DIO) and physical performance.

## Research Design and Methods

### Animals

C57BL6/J wild type (WT) and age matched C57BL6/J B2R knockout mice (B2R^-/-^) were obtained from the Universidade Federal de São Paulo, Brazil and from the Max-Delbrück-Center for Molecular Medicine, Berlin-Buch, Germany. All experiments reported have been conducted as stated in the National Institutes of Health guide for the care and use of laboratory animals (Institute of Laboratory Animal Resources, National Academy Press, Washington DC, 1996) and approved by a local committee. Animals were maintained on standard mouse chow at 22°C on a 12h light-dark cycle with *ad libitum* access to food and water. Food consumption and body weight were monitored weekly in individualized animals. In all experiments, 12- to 18-week-old males were used. Feed efficiency is the weight gain per ingested energy unit over a given period of time and was calculated at the end of the experimental period.

### HFD treatment

Three-month-old WT and B2R^-/-^ mice were fed either standard diet (10% kCal fat—cat#: D12450B) or high-fat diet (45% kCal fat—cat#:D12451) (Research Diets, New Brunswick, NJ) for 12 weeks. After treatment, mice were sacrificed for blood and tissue collection. The serum was separated for leptin and insulin quantification with ELISA kits (R&D Systems, Minneapolis, MN and Millipore, Billerica, MA, respectively). Tissues explants were weighed and frozen for protein, RNA or mtDNA extraction.

### Body composition analysis

Total body fat was estimated in WT and B2R^-/-^ mice using the GE-Lunar Radiation Corporation, model MD plus 73595 densitometer (Madison, WI, EUA). Whole-body densitometry was performed to measure the total body weight, fat content and lean mass weight. The equipment gives absolute and percentage values for lean and fat mass to the total mass of the studied animal. After sacrifice, the fat pad content was separated and weighed.

### Serum hormones measurements

After 8 weeks of HFD treatment, WT and B2R^-/-^ mice were submitted to an overnight fasting. Animals were euthanized and the blood serum separated by centrifugation for 15 minutes at 1500 g in room temperature. Leptin and insulin measurements were performed using the respective ELISA kit (R&D Systems Minneapolis, MN USA; Linco Research, St Charles, Missouri USA) protocols.

### Glucose and insulin tolerance tests

For intraperitoneal (IP) glucose tolerance tests (GTT), mice were injected with 10% glucose (D-glucose, Sigma, St. Louis, MO) in 0.9% saline (Teknova, Hollister, CA) at a 1 mg/kg dose. For IP insulin tolerance tests (ITT), the mice were injected with 100 mU of insulin (Humulin-R, Lilly, Indianapolis, IN) in 0.9% saline solution at a 1.0 U/kg dose. Blood glucose was measured before injection (time 0) and 15, 30, 60, 90 and 120 min after injection using a handheld glucometer. The results are shown as delta (glucose of each point, i.e. 15, 30, 60 and 90 minutes, divided by the glucose at time 0).

### Glucose uptake in isolated skeletal muscle

Mice were sacrificed and soleus muscles were carefully and quickly isolated, divided lengthwise, weighed and pre-incubated in Erlenmeyer flasks containing 3 mL Krebs-Ringer (containing 1% bovine serum albumin and 5mM glucose) under stirring with O_2_/CO_2_ atmosphere (95%/5%) for 30 minutes. After pre-incubation, muscles were transferred to other flasks containing 3 mL Krebs-Ringer medium plus 0.1 μCi/mL [U-^14^C]-D-glucose, 10,000 μU/mL insulin or without insulin (baseline). The muscles were incubated for 1 hour under the same conditions of preincubation, but the aeration with O_2_/CO_2_ gas mixture (95%/5%) occurred only during the first 15 minutes. [^14^C]-glycogen synthesis (as estimated by d-[^14^C]-glucose incorporation into glycogen) was determined as described by Leighton and Cooper [11] and the decarboxylation of d-[^14^C]-glucose and the uptake of 2-deoxy-d-[2,6-^3^H]-glucose were measured as previously described [12].

### Oxygen consumption

*In vivo* indirect open circuit calorimetry was performed in metabolic chambers. WT and B2R^-/-^ treated with control or HFD were randomly and alternatively placed into experimental chambers at 25°C ± 1 with free access to food and water. Constant airflow (0.5 L/min) was drawn through the chamber and monitored by a mass-sensitive flowmeter. To calculate oxygen consumption (VO_2_), carbon dioxide production (VCO_2_), and respiratory quotient (RQ: ratio of VCO_2_ to VO_2_), gas concentrations were monitored at the inlet and outlet of the scaled chambers.

### Mitochondrial content

Serial sections were cut transversely through the gastrocnemius muscle using a refrigerated (-20°C) cryostat (CTI Cryostat; IEC, Needham Heights, MA, USA). Sections were stained for succinate dehydrogenase (SDH) and cytochrome c oxidase (COX) to determine the activity of oxidative enzymes (SDH) and mitochondrial integrity (COX) (5μm sections). SDH staining was performed by incubating sections for 1 h at 37°C in a 0.2 M sodium phosphate buffer containing 0.1 M succinic acid and 1.2 mM nitro-blue tetrazolium. Sites of SDH activity were coloured blue. COX staining was performed by incubating sections for 1 h in a 50 mM Tris/HCl buffer (pH 7.6) containing 0.22 M sucrose, 14 mM 3,3′-diaminobenzidine tetrahydrochloride, 80 μM cytochrome c and 1300 U catalase. Sites of COX activity were coloured brown. The optical density of SDH staining was determined after 6 min of reactivity for all samples and stained sections captured in full color using bright-field light microscopy. Digitally captured images with a minimum of four fields of view per muscle cross-section were analyzed. The bright-field images of SDH reactivity were converted *post hoc* to gray scale values. The mean optical density of the SDH-raised signal per individual fiber was determined by averaging the optical density measured in every pixel in the cell, corrected for the mean optical density of the background stain measured in a field of view containing no muscle fibers. SDH activity is, therefore, expressed as optical density (o.d.).

### Superoxide anion

DHE (dihydroethidium, Dako) is cell permeable and reacts with superoxide (O_2_•−) to form ethidine, which in turn intercalates with DNA, providing nuclear fluorescence. To get rid of the differences in cellular densities, DAPI (4′,6-diamidino-2-phenyindole, Sigma) was used to determine cellular density. Unfixed fresh-frozen gastrocnemius muscle slides were fixed in acetone for 10 min. The slides were incubated in a light-protected humidified chamber at room temperature with a 5 μM DHE–0.25 μg/ml DAPI solution for 5 min. The slides were then rinsed in PBS (Dako) and mounted with a fluorescent mounting medium containing an anti-fading agent (Dako). To confirm the specific detection of O_2_•− of DHE, several slides were incubated with 300 IU/ml superoxide dismutase (Sigma) before DHE incubation. The slides were immediately analyzed with a computer-based digitizing image system (Microvision, Evry) using a fluorescent microscope (Eclipse 600, Nikon, Champigny-Sur-Marne) connected to a video camera (Tri CCD, Sony). Ethidine fluorescence was detected with a 510–560 nm excitation and 590 nm emission filters. DAPI fluorescence was detected with 330–380 nm excitation and 420 nm emission filters. Automatic computer-based analysis was performed with the same threshold for all sections (×200 magnification). The number of nuclei per section was determined (DAPI staining). Muscle surface was delimited and fluorescence emission analyzed (DHE staining). The results were expressed as the ethidine fluorescence/nucleus as previously described [13].

### Gene expression

RNA and DNA were extracted according to manufactures protocol (Trizol and Qiagen). The SYBR Green System was used for quantification of gene expression. To this end, reactions were performed in a final volume of 20 μL containing 30–100 ng of cDNA or mtDNA, 10 μL of SYBR Green Universal PCR Master Mix 2x and 1 μL of each sense and antisense oligo (10 mM each). The cycling protocol was followed according to the determination of the unit standard 7500 from Applied Biosystems, including the dissociation curve. mRNA reactions were conducted with the following primers: *β-actin* 5’-CTGGCCTCACTGTCCACCTT-3’, 5’-GGACTCATCGTACTCCTGCTT-3’; *UCP3* 5’-TCTTGTGATGTTGGGCCAAG-3’, 5’-TTCAAGCCATGATACGCCTG-3’; *PPARgama* 5’-CCACCAACTTCGGAATCAGCT-3’, 5’-AGGAATGCGAGTGGTCTTCCA-3’; *PGC1beta* 5’-GAGGAGTCCCTTCCCTCATC-3’, 5’-TCCTCGAAGGTTAAGGCTGA-3’; *PGC1alpha* 5’-TGCGTGTGTGTATGTGTGTGTG-3’, 5’-CCTTGTTCGTTCTGTTCAGGTG-3’; *LCAD* 5’-CATATTCCCCCAGGACATTG-3’, 5’-CACAATTGCCTCTATGTGCATT-3’, *CPT1* 5’-CTTCCATGACTCGGCTCTTC-3’, 5’-AGCTTGAACCTCTGCTCTGC-3’, *SCD1* 5’-GTATCGCCCCTACGACAAGA-3’, 5’-GCGTGATGGTAGTTGTGGAA-3’. For the expression analysis of mtDNA the following oligos were used: *mt-CO1* 5'-CCCAATCTCTACCAGCATC-3', 5'-GGCTCATAGTATAGCTGGAG-3', *mt-Cytb* 5'-TTCTGAGGTGCCACAGTTATT-3', 5'-GAAGGAAAGGTATTAGGGCTAAA-3', *mt-Nd1* 5'-AATGGCCATAGCCTTCCTAACAT-3', 5'-GGCGTCTGCAAATGGTTGTAA-3', *mt-H19* 5'-GTACCCACCTGTCGTCC-3', 5'-GTCCACGAGACCAATGACTG-3'. Standard curves for each pair of oligo and cDNA sample group were made to establish the efficiency of these reactions. For the analysis of gene expression only reactions with high efficiency (> 95%) were used. This allowed us to use the 2^-ΔCt^ parameter for expressing arbitrary value of relative gene expression for each sample, using as endogenous control β-actin gene (mRNA) and nuclear encoded H19 gene (mtDNA) from the sample itself.

### Graded treadmill exercise test

Exercise performance was evaluated using a graded treadmill exercise protocol for mice as previously described [14]. Briefly, after being adapted to treadmill exercises over a week (10 min of exercise session), mice were placed in the exercise streak and allowed to acclimatize for at least 30 min. Then, the exercise intensity was increased by 3 m/min (6–33 m/min) every 3 min at 0% grade until exhaustion.

### Swimming exhaustive test

Time to exhaustion was evaluated in a swimming apparatus especially planned for exercise training of mice [15, 16]. The system consists of water glass tanks of different dimensions. The outer tank displays 95 cm in length, 50 cm in width and 45 cm in height. The inner tank is divided into 10 lanes with a surface area of 15 x 15 cm per lane and a depth of 35 cm to allow individual training. To prevent floating during the swimming session, tubes were connected to an air pump system producing water bubbling, and additional weight (3% of BW) was added to the tails. A heating system kept the water temperature between 30 and 32°C.

### Muscle fiber-typing and cross-sectional area

Soleus muscle was harvested from WT and B2R^-/-^ mice, immediately frozen in melting isopentane, and stored in liquid nitrogen. Frozen muscles were cut into 10-μm cross sections from the proximal to distal region using a cryostat (Criostat Mícron HM505E, Walldorf, Germany). Muscle sections were then incubated for myofibrillar ATPase activity after alkali (mATPase, pH 10.3) or acid pre-incubation (mATPase, pH 4.6) as previously described [17]. The myosin ATPase reaction was used to identify the muscle fiber type. Type I fibers reacted deeply after acid preincubation at pH 4.6, and lightly after formaldehyde pretreatment and alkali preincubation at pH 10.3. The inverse occurred with type II muscle fibers. Fiber typing and fiber cross-sectional area were evaluated in whole muscles at 200x magnification and further analyzed on a digitizing unit connected to a computer (Image Pro-plus, Media Cybernetic, Silver Spring, MD, USA). The total number of each fiber type was counted in order to calculate the numerical fiber type composition (I, IIA, and Intermediary). The fibers that could not be clearly identified as I or IIA were classified as intermediary (they presented light stain at pH 10.3 and a small area). The cross-sectional area of each fiber type was measured for further calculation of averaged fiber cross-sectional area. All analyzes were conducted by a single observer, blinded to mice’s identity.

### Statistical analysis

All values are expressed as mean ± SEM. Statistical analyses were carried out using two-tailed Student's unpaired t test to compare two independent groups, or ANOVA followed by Bonferroni's test to compare more than two. Significance was rejected at p>0.05.

## Results

### Body weight and composition after high-fat diet

**Fig 1:**
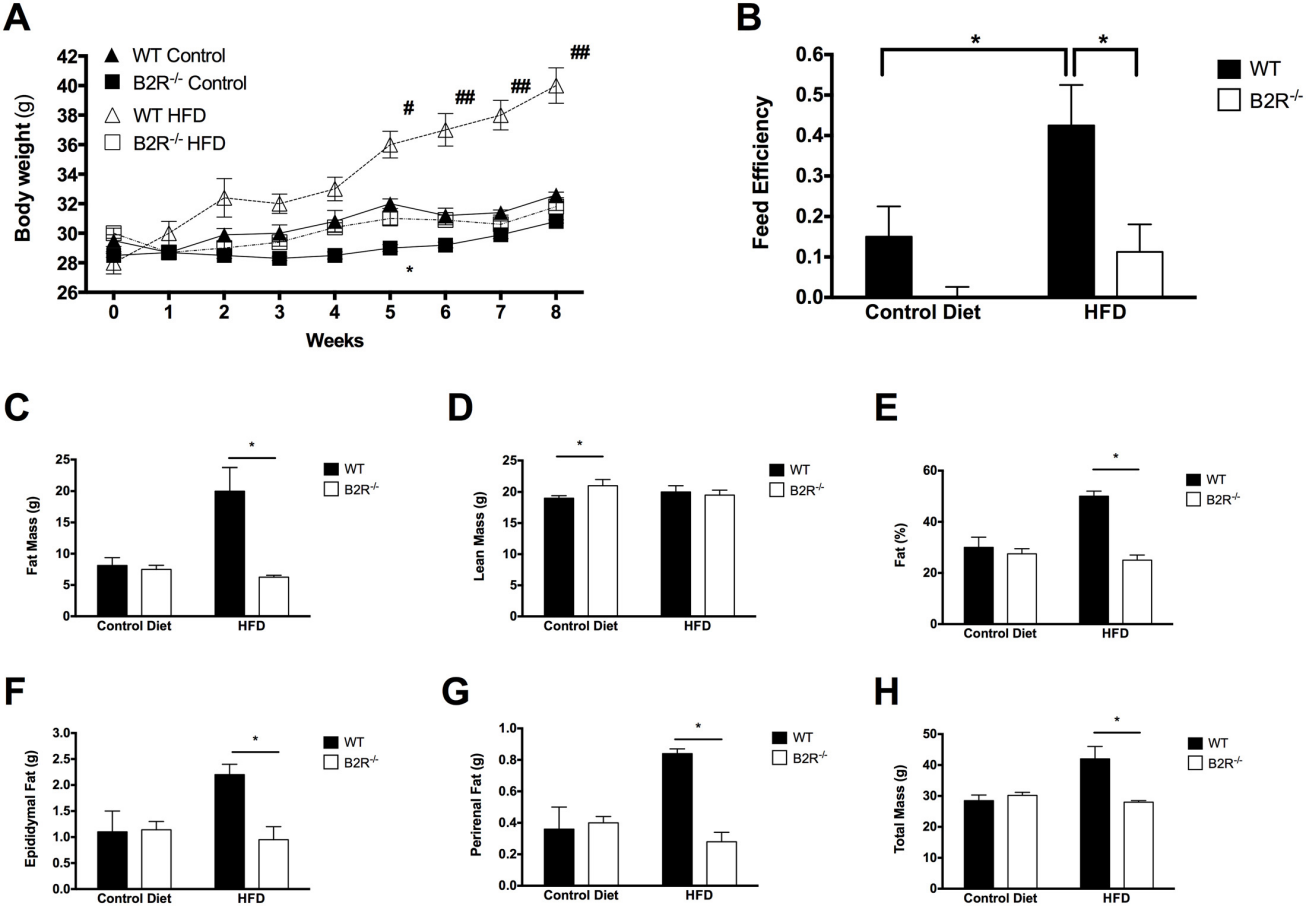
B2R^-/-^ is protected from obesity. **(A)** Total body weight of wild type (WT) or B2 knockout mice (B2R^-/-^) under control diet (Control) or high-fat diet (HFD) (*n* = 5 each). Obesity was induced after 8 weeks of HFD treatment. **(B)** Feed efficiency of WT and B2R^-/-^ mice during 8 weeks of HFD treatment. Body composition analysis showing **(C)** fat mass, **(D)** lean mass and percent body fat **(E)** of WT and mice after 8 weeks of HFD treatment. **(F)** Epididmal and **(G)** perirenal fat pad depots and **(H)** total mass after 8 weeks of HFD treatment. (*n* = 5 for each group) (*P<0.05; ^*#*^ *P<0.05*, ^*##*^ *P<0.01) Data are presented as mean ± S*.*E*.*M*.

To assess the contribution of the kinin B2 receptor to the regulation of adiposity in mice, we analyzed body weight, feed efficiency and white adipose tissue mass in control and HFD fed B2R^-/-^ and wild type (WT) animals. Under control diet, B2R^-/-^ mice presented normal body weight. Wild type animals displayed an expected elevation in body weight and adipose mass after HFD, whereas B2R^-/-^ mice were remarkably refractory to the fat regimen (Fig 1A). More notably, feed efficiency (grams of weight gained/food consumed) was decreased markedly in the absence of kinin B2 receptor (Fig 1B), suggesting increased energy expenditure. The profile of animal body mass subjected to different types of diet was assessed. Using body densitometry we have obtained the fat mass (Fig 1C), lean mass (Fig 1D) and percentage of body fat (Fig 1E) of each animal. As a result, we observed a significant difference in epididymal (Fig 1F), perirenal (Fig 1G) fat content and total body weight (Fig 1H) of B2R^-/-^ after the HFD treatment.

### Blood leptin and insulin levels, glucose and insulin tolerance test and glucose uptake in skeletal muscle

**Fig 2:**
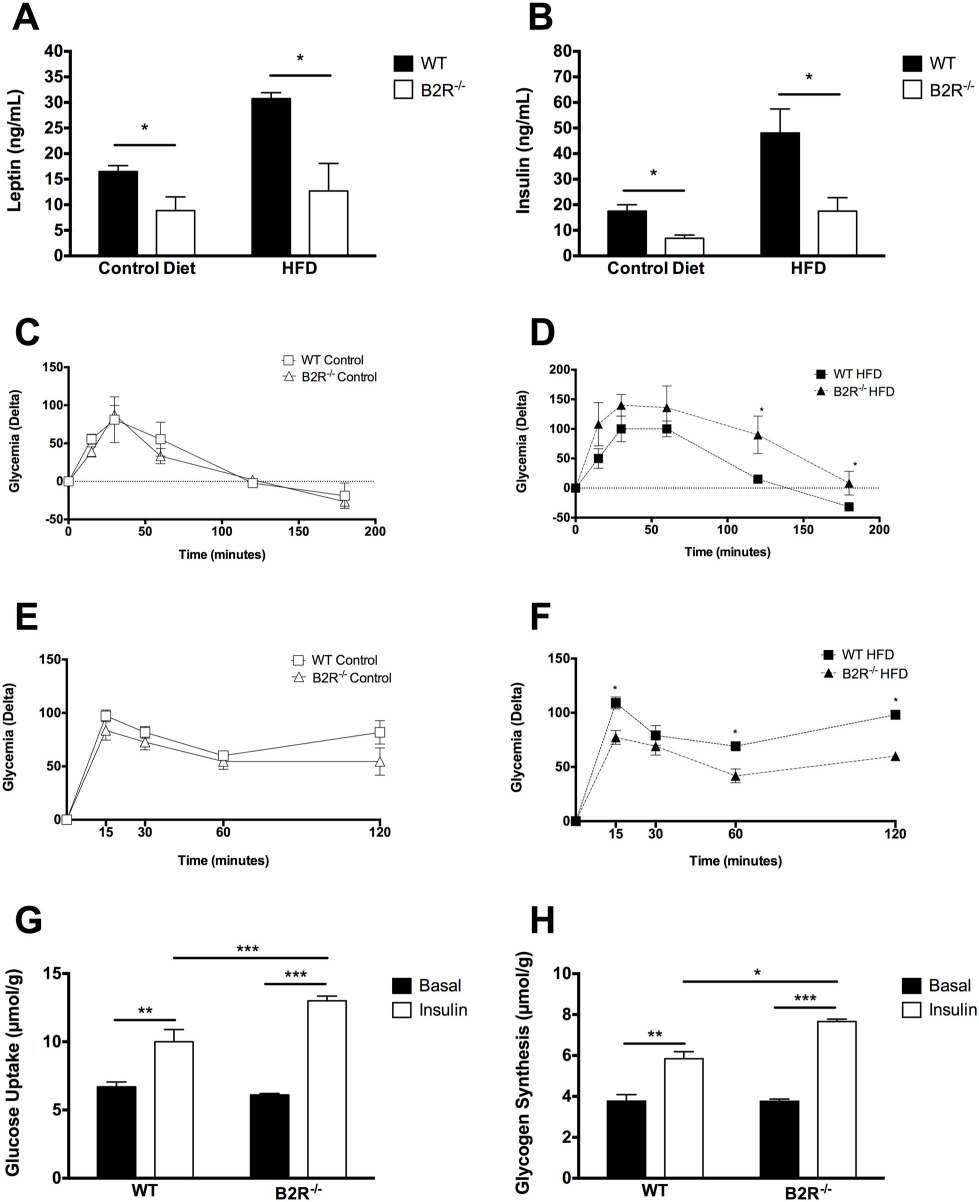
Leptin and Insulin levels, GTT and ITT and skeletal muscle glucose uptake. Serum leptin **(A)** and insulin **(B)** levels measured after 8 weeks of HFD treatment in WT and B2R^-/-^ mice under control (Control) or high-fat diet (HFD). **(C)** Glucose tolerance test of WT and B2R^-/-^ under control diet and **(D)** high-fat diet **(E)** Insulin tolerance test of WT and B2R^-/-^ under control diet and **(F)** high-fat diet **(G)** Glucose uptake and **(H)** glycogen synthesis in skeletal muscle of 3-month-old WT and B2R^-/-^ mice incubated without (filled bars) or with (open bars) 10,000 μU/mL of insulin for 60 min. (*n* = 5 for each group). (*P < 0.05; **P<0.*005*, ***P<0.*001) Data are presented as mean ± S*.*E*.*M*.

We measured serum leptin (Fig 2A) and insulin (Fig 2B) levels and found a significant decrease in leptin and insulin content in B2R^-/-^ mice when compared to WT mice. Notably, HFD did not lead to an increase of leptin and insulin levels in the B2R^-/-^ mice. These results demonstrate that B2R^-/-^ mice have reduced adiposity and are strongly resistant to diet-induced obesity and hyperleptinemia. Glucose tolerance was measured in WT and B2R^-/-^ under control diet (Fig 2C) and HFD (Fig 2D) and no difference was observed between groups under control diet but significant glucose intolerance in B2R^-/-^ when submitted to HFD. Despite that, insulin sensitivity was not different under control diet (Fig 2E), but B2R^-/-^ presented higher insulin sensitivity under HFD when compared to WT at the same food regimen (Fig 2F). Fig 2G shows that glucose uptake in muscle of WT and B2R^-/-^ mice was not different at basal level, but after insulin stimulation knockout animals had a higher glucose uptake. The synthesis of glycogen (Fig 2H) was also measured, and as well as for glucose uptake, the basal production of glycogen was not significantly different between the two groups. However, after the addition of insulin, B2R^-/-^ animals showed a significant increase in glycogen levels when compared to WT.

### Energy expenditure, mitochondrial activity and superoxide anion

**Fig 3:**
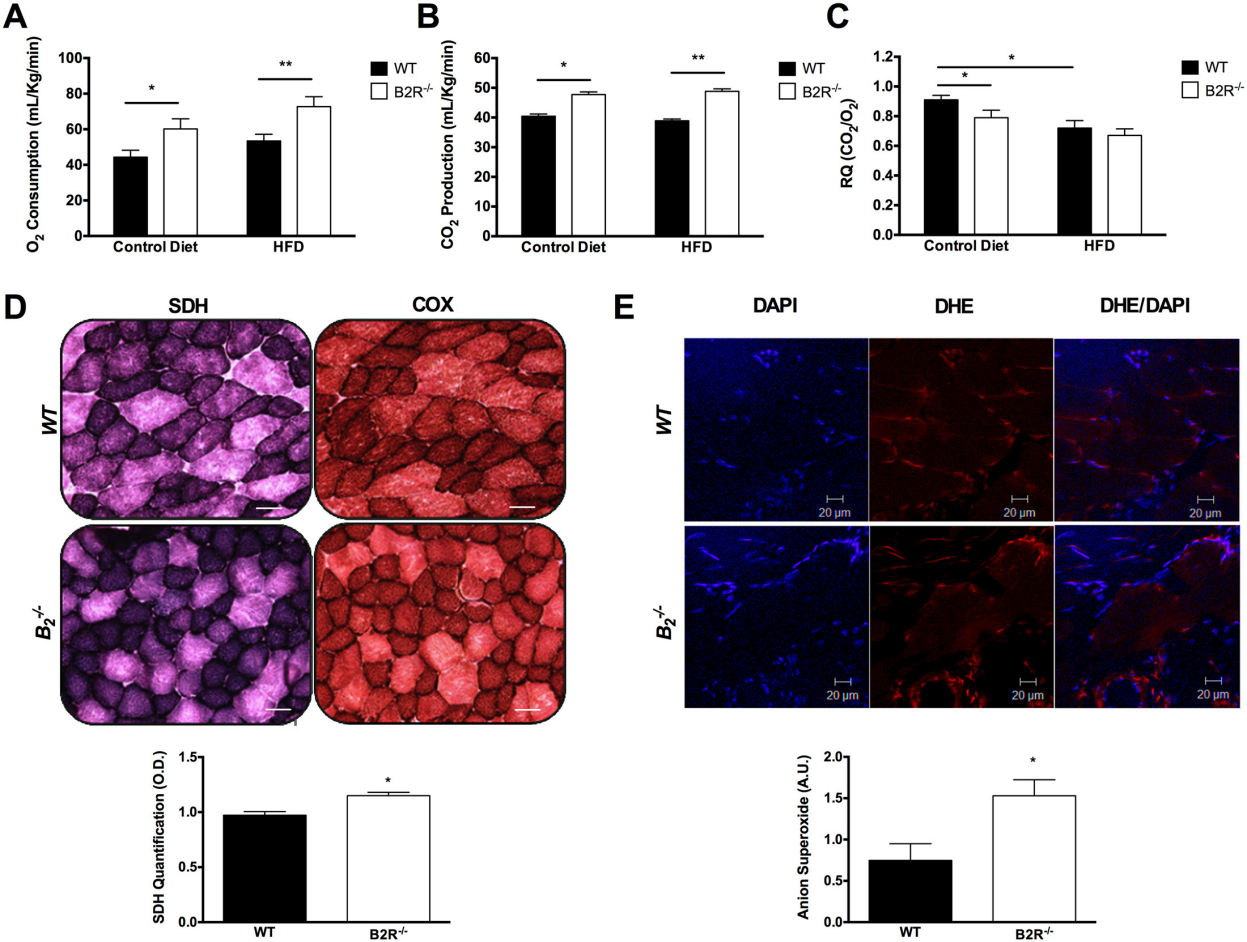
Indirect calorimetry and mitochondrial activity. **(A)** Oxygen consumption and **(B)** CO_2_ production in WT or B2R^-/-^ mice fed an HFD for 8 weeks (n = 5 each). **(C)** Respiratory quotient ratio in WT or B2R^-/-^ mice fed an HFD for 8 weeks. (n = 5 each). **(D)** Histochemical analysis of SDH and COX staining in skeletal muscle of 3-month-old WT or B2R^-/-^ mice (n = 5) Scale bar = 50μm. **(E)** Dihydroethidium (DHE) and 4’,6-diamidino-2-phenylindole dihydrochloride (DAPI) colocalization. Representative images of gastrocnemius muscle in 3-month-old mouse showing fluorescent labeling of DAPI (blue) in and DHE (red) at the corresponding location and focal plane, with merged pictures, showing colocalization (pink). Scale bar = 20μm. (*P < 0.05; **P<0.005) *Data are presented as mean ± S*.*E*.*M*.

To determine the metabolic profile of the experimental groups treated with different types of diet, the animals were subjected to analysis of O_2_ consumption (Fig 3A) and CO_2_ production (Fig 3B). Before and after the diet treatment we found a significant difference in oxygen consumption of animals B2R^-/-^. Due to the higher oxygen consumption, we also found a higher carbon dioxide production, a result of the increase in the respiratory process in mice lacking B2 receptor. The respiratory quotient (RQ) values for the wild type and knockout mice were obtained by dividing the volumes of CO_2_ released by the amount of O_2_ consumed. We found that the knockout animals have significantly lower RQ than that of WT mice under normal conditions (Fig 3C).

The mitochondrial activity was obtained by quantification of an enzyme component of the electron transport chain, Succinate Dehydrogenase (SDH). The enzyme cytochrome C oxidase (COX) activity has also been examined to validate the experiment and analysis of mitochondrial activity (Fig 3D). As a result of a higher mitochondrial activity, B2R^-/-^ mice have higher superoxide anion levels in this tissue compared to wild type (Fig 3E).

### Gene expression

**Fig 4:**
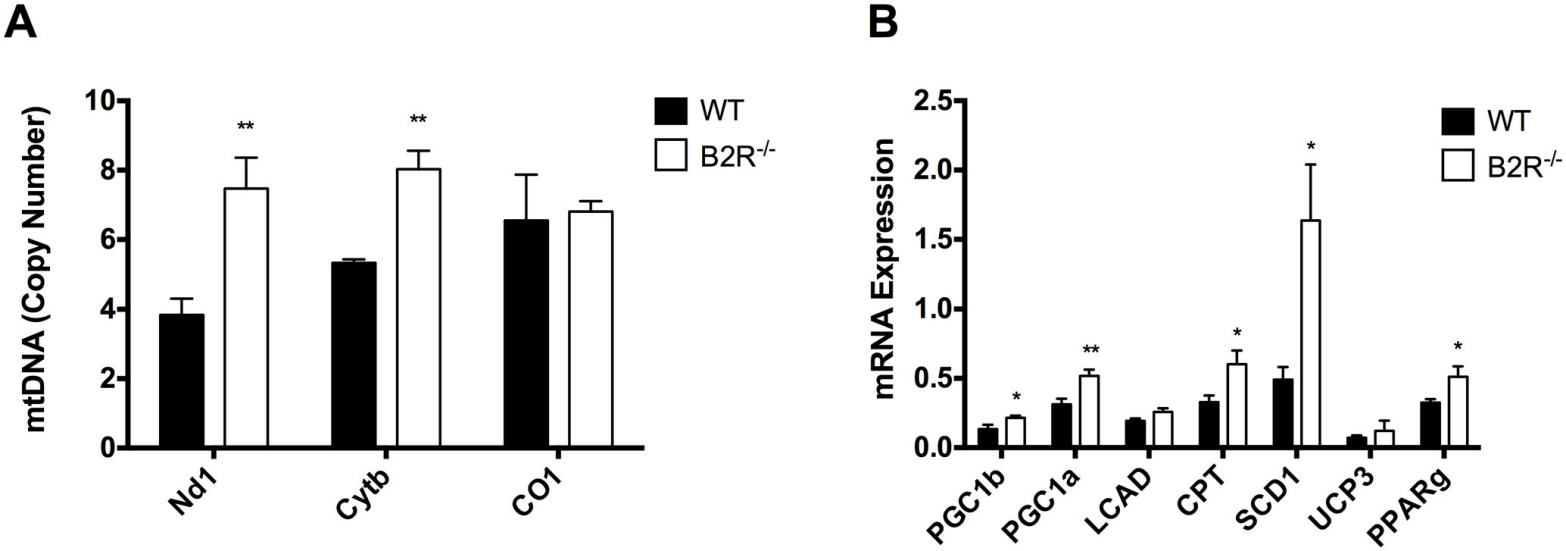
Mitochondrial biogenesis. **(A)** Mitochondrial DNA quantification in skeletal muscle of 3-month-old WT or B2R^-/-^ mice (n = 5 each). **(B)** Expression of genes related to mitochondrial biogenesis (PGC1b; PGC1a; LCAD; SCD1; UCP3; PPARg) and OxPhos (CPT) in skeletal muscle of 3-month-old WT or B2R^-/-^ mice (*n* = 7 each). (*P < 0.05; **P<0.*005) Data are presented as mean ± S*.*E*.*M*.

To verify whether B2R^-/-^ mice have a higher amount of mitochondria in muscle, mitochondrial DNA was extracted and quantified by real time PCR (Fig 4A). The chart shows increased levels of muscular NADH dehydrogenase 1 (mt-Nd1) and cytochrome b (mt-Cytb) in B2R^-/-^. This result, associated with mitochondrial activity shown in the previous section, indicates that the B2R^-/-^ animals have a higher mitochondrial activity and higher amount of mitochondria in skeletal muscle.

To elucidate the possible mechanism of action of B2 receptor in glucose and fatty acid metabolism and mitochondrial biogenesis, we measured the mRNA expression of PGC1 alpha and beta, LCAD, CPT, SCD1, UCP3 and PPAR gamma in the muscle of WT and B2R^-/-^ mice. We observed an increase in the expression of the mitochondrial genes PGC1 alpha, PPAR gamma and of the fatty acid metabolism genes CPT and SCD1, which can contribute to the phenotype presented by B2R^-/-^ (Fig 4B).

### Muscle characteristics

**Fig 5:**
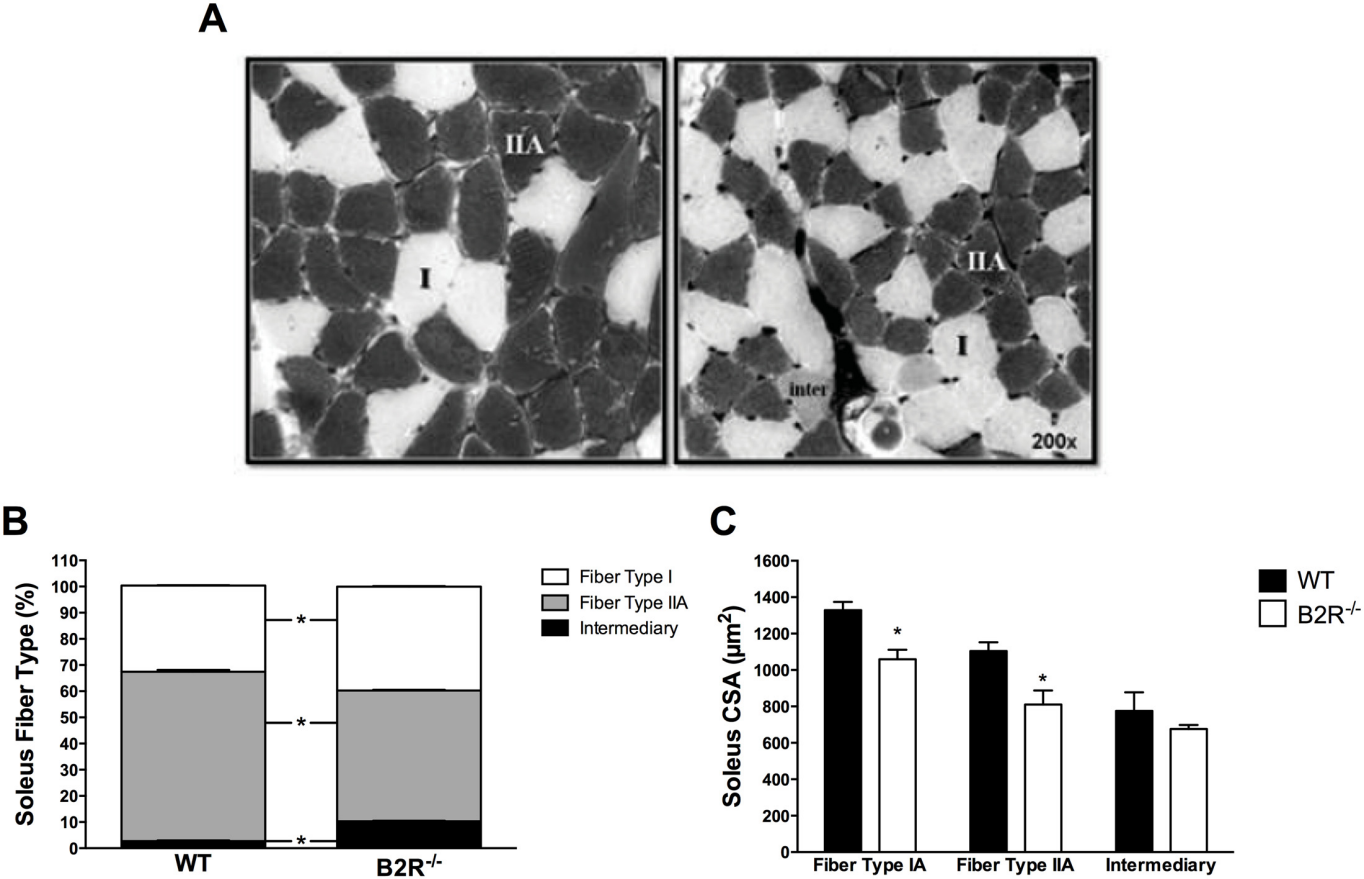
Fiber type characterization in skeletal muscle. **(A**) Example of transverse soleus sections from 3-month-old WT and B2R^-/-^ mice, with histochemical staining for myosin ATPase, preincubated at pH 10.3. Type I fibers; type IIA fibers and intermediary fiber are indicated. **(B)** Soleus fiber type percentage. **(C)** Media cross-sectional area (CSA) of soleus muscle from WT and B2R^-/-^. (*n* = 5 each). (*P < 0.*05) Data are presented as mean ± S*.*E*.*M*.

The soleus muscle of B2R^-/-^ mice display an increased type I and intermediary, but a decreased type IIA fiber percentage in comparison to their age-matched WT counterparts, suggesting a fiber type shift (IIA to I) (Fig 5A and 5B). Cross-sectional area (CSA) of fibers type I and IIA in soleus muscle were smaller in B2R^-/-^ than in WT mice (Fig 5C). There was no difference in the cross-sectional area of intermediary fibers between B2R^-/-^ and WT animals.

### Graded treadmill maximal test

**Fig 6:**
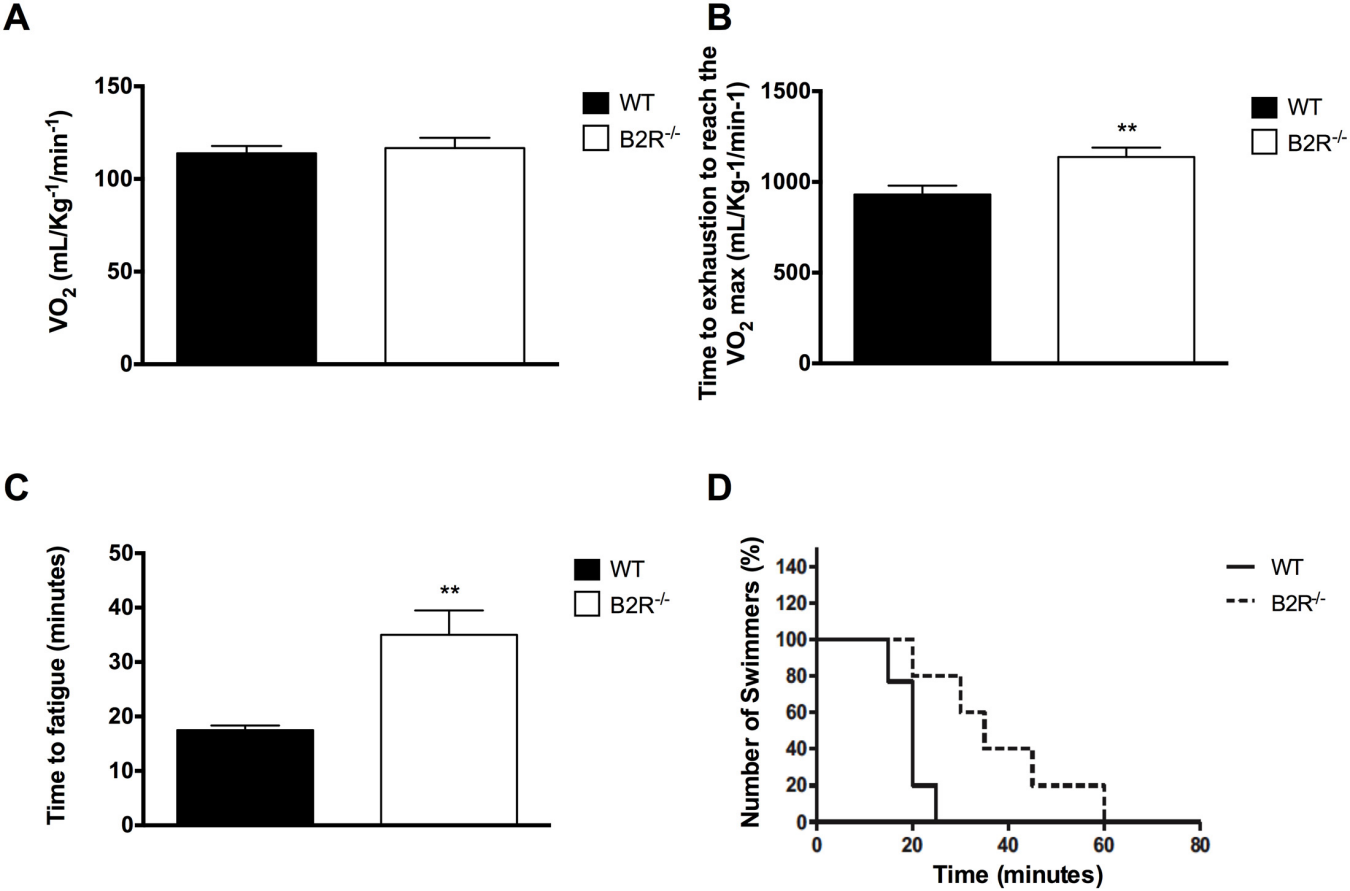
Exercise performance. **(A)** Maximum VO_2_ and **(B)** time to exhaustion of WT or B2R^-/-^ mice on a metabolic treadmill. **(C)** Time to fatigue after swimming test and **(D)** percentage of swimmers in 3 month-old WT and B2R^-/-^ mice (n = 5 each). (**P<0.*005) Data are presented as mean ± S*.*E*.*M*.

B2R^-/-^ mice and their WT counterparts displayed similar maximal VO_2_ (VO_2_^max^) in graded treadmill running test (Fig 6A). However, the effort expended at the same percentage of VO_2_^max^ was lower in B2R^-/-^ when compared with the WT mice (Fig 6B), which suggests that B2R^-/-^ mice displayed better metabolic efficiency during the test than WT counterparts.

### Swimming exhaustive test

When submitted to swimming exhaustive test, B2R^-/-^ mice were able to swim for a longer time in comparison to their WT counterparts (Fig 6C). Only 20% of WT mice swam for more than 20 minutes, as opposed to 80% of B2R^-/-^ mice, which were able to swim twice in this time. Also 20% of B2R^-/-^ mice remained swimming up to 60 minutes (Fig 6D).

## Discussion

We have previously demonstrated the relationship between kinins and metabolic processes such as insulin and glucose homeostasis and obesity [18–20]. Also, we showed that B2R regulates skeletal muscle mass by altering myostatin gene expression [21]. Due to these factors, we hypothesized that B2R could have a role in metabolic disorders and physical performance and therefore submitted B2R^-/-^ mice to a high fat diet treatment and physical tests. The B2R^-/-^ displayed a remarkable resistance to diet induced obesity (DIO) when compared to control wild type mice. In addition, they displayed higher energy consumption with preference for fatty acid as energy source (as demonstrated by RQ), which prompted us to aim the skeletal muscle and its role in oxidative metabolism. As mentioned herein, total SM mass highly influences body metabolism. Thus, the increased muscle mass presented by B2R^-/-^ (under standard chow) is in accordance with the higher rate of energy expenditure observed in the present study. Also reinforcing the involvement of KKS in muscle mass control, it was recently demonstrated that the increased expression of kinin B1 receptor (B1R) in an androgen-sensitive model of SM atrophy was associated to a reduction of C2C12 myocytes [22]. The mechanism involved in this effect depends on the ability of B1R to regulate specific E3-ligases atrogin-1 and MuRF-1 influencing the regulation of SM proteolysis.

β-oxidation in mitochondria is the main process whereby fatty acids are metabolized to generate ATP. The lower RQ observed in B2R^-/-^ suggests that this pathway is mostly used to produce energy, and supporting this finding, mitochondrial oxidative phosphorylation (mtOxPhos) genes are upregulated in skeletal muscle of B2R^-/-^ mice. In addition, mice lacking kinin B2R present higher activity as well as a higher number of mitochondria in skeletal muscle, showing an increase in anion superoxide, oxidative genes and also mitochondrial genes.

Mitochondria functional status is associated both with diseases and physical performance. For example, mitochondrial dysfunction and fat accumulation in muscle has been implicated with type 2 diabetes, insulin resistance and obesity [23–25]. Furthermore, it was reported that the SM fiber distribution in humans was associated with metabolic syndrome [26]. Specifically, the proportion of type I fibers in *vastus lateralis* muscle was correlated with the severity of insulin resistance [26]. B2R^-/-^ mice present lower levels of blood insulin and leptin probably as a consequence of the lower amount of white adipose tissue. Duka et al. (2001) showed that the deletion of B2 receptor causes glucose intolerance and Schweitzer et al. (2011) exhibited that B2R is not essential for the effects of glucose uptake in insulin-stimulated mouse soleus muscle in B6/129SvF2 strain [6, 27]. Here, B2R^-/-^ presented glucose intolerance only when submitted to HFD, but higher sensitivity to insulin after HFD probably caused by the decreased body fat. We also observed higher insulin sensitivity in skeletal muscle under normal conditions, which accounts for 80% of whole body insulin-mediated glucose utilization [28]. On the other hand, it is well known that regular physical exercise increases SM capacity to oxidize glucose and fatty acids [29], enhance physical performance and the capacity to expend energy [30]. The peroxisome proliferator-activated receptors (PPARs) class of transcription factors controls transport and metabolism of these substrates and exercise adaptation is mediated by the stimulation of PPAR and mitochondrial genes [31]. In this sense, the increase observed in gene expression of PPARy, CPT, SCD1 Nd-1, Cytb as a consequence of B2R absence is very similar to the effects induced by chronic aerobic exercise in SM.

Deletion of B2 receptor in mice also had a profound impact on skeletal muscle functionality. B2R^-/-^ mice displayed reduced soleus cross-sectional area of fiber types I and IIA when compared to their WT counterparts. The reduced cross sectional area in these type of fibers of B2R^-/-^ mice was paralleled by increased percentage of type I (oxidative) and intermediary fibers with a reduction in type IIA (oxidative/glycolytic) fibers, which suggest a shift from type IIA to I fiber and pro oxidative metabolism. These results are in line with training specifically to marathon running, which also promotes a reduction of type I fiber diameter associated with increased contractile properties and improved oxidative capacity [31].

In the present study, the higher percentage of oxidative skeletal muscle fibers in B2R^-/-^ mice was coupled with an increased aerobic performance. This feature was not affected by exercise mode since aerobic performance was increased both in treadmill and swimming, the most widely used tests for evaluating aerobic capacity in animal studies [32]. Interestingly, the higher aerobic performance observed in B2R^-/-^ mice was achieved even with similar VO_2_^max^ observed in B2R^-/-^ and WT mice. To better understand this response, one needs to notice that the same absolute values for VO_2_^max^ were achieved at higher work rate in B2R^-/-^ when compared to WT mice, which means that B2R^-/-^ is able to reach the same level in the graded treadmill exercise test using a lower percentage of aerobic power. The B2R absence also affected the swimming test performance shown by the longer time to exhaustion. Whereas approximately 80% of WT mice were exhausted after 20 minutes of swimming, at least 40% of B2R^-/-^ mice swam twice longer and 20% of B2R^-/-^ mice could remain swimming for up to 60 minutes. Furthermore, the mean time until exhaustion was approximately 75% higher in B2R^-/-^ when compared to WT mice. These data give support for the hypothesis that B2R^-/-^ is metabolically more efficient when compared to WT mice.

Of note, the KKS also seems to be involved in the adaptive response to anaerobic training. It was demonstrated that the +9/-9 polymorphism of B2R was associated with SM response to resistance training [33]. Specifically, the expression of B2R is regulated by a common repeat sequence variation of 9bp (+9/-9 alleles) in exon 1 of the B2R gene [34]. In this context, the -9 allele is associated with a higher expression level of B2R [33]. In the mentioned study, it was demonstrated that individuals homozygous for the -9 allele presented a higher triceps brachii hypertrophy in response to chronic training than individuals with one or two +9 alleles [35].

Taken together, all these evidences and our findings give support to the involvement of the KKS in skeletal muscle trophicity and fiber type distribution, which ultimately seems to determine the energy substrate fate and affects aerobic performance during exercise. Further, an eventual role of KKS in metabolic diseases and disorders that affect muscle mass structure should also be investigated in future studies.

## References

[1] Mori MA, Araújo RC, Reis FC, Sgai DG, Fonseca RG, Barros CC, et al Kinin B1 receptor deficiency leads to leptin hypersensitivity and resistance to obesity. Diabetes. 2008;57(6):1491–500. 10.2337/db07-1508 18332096

[2] Mori MA, Merino VF, Bascands JL, Schanstra JP, Zollner RL, Villela CA, et al Role of the kinin B1 receptor in insulin homeostasis and pancreatic islet function. Araújo RC, Biol Chem. 2006;387(4):431–6. 10.1515/BC.2006.057 16606341

[3] Dietze GJ, Wicklmayr M, Rett K, Jacob S, Henriksen EJ. Potential role of bradykinin in forearm muscle metabolism in humans. Diabetes. 1996;45 Suppl 1:S110–4. 8529790 10.2337/diab.45.1.s110

[4] Miyata T, Taguchi T, Uehara M, Isami S, Kishikawa H, Kaneko K et al Bradykinin potentiates insulin-stimulated glucose uptake and enhances insulin signal through the bradykinin B2 receptor in dog skeletal muscle and rat L6 myoblasts. European journal of endocrinology / European Federation of Endocrine Societies. 1998;138(3):344–52. 9539311 10.1530/eje.0.1380344

[5] Kishi K, Muromoto N, Nakaya Y, Miyata I, Hagi A, Hayashi H et al Bradykinin directly triggers GLUT4 translocation via an insulin-independent pathway. Diabetes. 1998;47(4):550–8. 9568686 10.2337/diabetes.47.4.550

[6] Duka I, Shenouda S, Johns C, Kintsurashvili E, Gavras I, Gavras H. Role of the B(2) receptor of bradykinin in insulin sensitivity. Hypertension. 2001;38(6):1355–60. 11751717 10.1161/hy1201.096574

[7] de Picoli Souza K, Batista EC, Silva ED, Reis FC, Silva SM, Araujo RC et al Effect of kinin B2 receptor ablation on skeletal muscle development and myostatin gene expression. Neuropeptides. 2010;44(2):209–14. 20045188 10.1016/j.npep.2009.12.001

[8] Lee B, Shao J. Adiponectin and energy homeostasis. Reviews in endocrine & metabolic disorders. 2014;15(2):149–56. 10.1007/s11154-013-9283-3 24170312 PMC4006341

[9] Gollnick PD, Matoba H. The muscle fiber composition of skeletal muscle as a predictor of athletic success. An overview. The American journal of sports medicine. 1984;12(3):212–7. 6204545 10.1177/036354658401200309

[10] Guderley H. Locomotor performance and muscle metabolic capacities: impact of temperature and energetic status. Comparative biochemistry and physiology Part B, Biochemistry & molecular biology. 2004;139(3):371–82. 10.1016/j.cbpc.2004.04.001 15544962

[11] Leighton B, Cooper GJ. Pancreatic amylin and calcitonin gene-related peptide cause resistance to insulin in skeletal muscle in vitro. Nature. 1988; 335:632–635. 3050530 10.1038/335632a0

[12] Hirabara SM, Folador A, Fiamoncini J, Lambertucci RH, Rodrigues CF Jr, Rocha MS, et al Fish oil supplementation for two generations increases insulin sensitivity in rats. J Nutr Biochem. 2013;24:1136–45. 10.1016/j.jnutbio.2012.08.014 23246156

[13] Calió ML, Marinho DS, Ko GM, Rodrigues R, Carbonel AF, Oyama LM, et al Transplantation of bone marrow mesenchymal stem cells decreases superoxide, apoptosis and lipid peroxidation in brain of a spontaneously stroke model. Free Radical Biology and Medicine. 2014;70:141–54. 10.1016/j.freeradbiomed.2014.01.024 24525001

[14] Girgenrath S, Song K, Whittemore LA. Loss of myostatin expression alters fiber-type distribution and expression of myosin heavy chain isoforms in slow- and fast-type skeletal muscle. Muscle & nerve. 2005;31(1):34–40. 10.1002/mus.20175 15468312

[15] Batista EC, Ramalho JD, Reis FC, Barros CC, Moraes MR, Pesquero JL et al Swimming training exacerbates pathological cardiac hypertrophy in kinin B2 receptor-deficient mice. International immunopharmacology. 2008;8(2):271–5. 10.1016/j.intimp.2007.08.029 18182239

[16] Wasinski F, Bacurau RF, Moraes MR, Haro AS, Moraes-Vieira PM, Estrela GR et al Exercise and caloric restriction alter the immune system of mice submitted to a high-fat diet. Mediators of inflammation. 2013;2013:395672 10.1155/2013/395672 23576853 PMC3610381

[17] Williams AG, Rayson MP, Jubb M, World M, Woods DR, Hayward M et al The ACE gene and muscle performance. Nature. 2000;403(6770):614 10.1038/35001141 10688186

[18] Fonseca RG, Sales VM, Ropelle E, Barros CC, Oyama L, Ihara SS et al Lack of kinin B(1) receptor potentiates leptin action in the liver. Journal of molecular medicine. 2013;91(7):851–60. 10.1007/s00109-013-1004-6 23385644

[19] Barros CC, Haro A, Russo FJ, Schadock I, Almeida SS, Reis FC et al Bradykinin inhibits hepatic gluconeogenesis in obese mice. Laboratory investigation; a journal of technical methods and pathology. 2012;92(10):1419–27. 10.1038/labinvest.2012.105 22868909

[20] Barros CC, Haro A, Russo FJ, Schadock I, Almeida SS, Ribeiro RA et al Altered glucose homeostasis and hepatic function in obese mice deficient for both kinin receptor genes. PloS one. 2012;7(7):e40573 10.1371/journal.pone.0040573 22829877 PMC3400662

[21] Kopelman PG. Obesity as a medical problem. Nature. 2000;404(6778):635–43. 10.1038/35007508 10766250

[22] Parreiras ESLT, Reis RI, Santos GA, Pires-Oliveira M, Pesquero JB, Gomes MD et al The kinin B1 receptor regulates muscle-specific E3 ligases expression and is involved in skeletal muscle mass control. Clinical science. 2014;127(3):185–94. 10.1042/CS20130358 24498923

[23] Petersen KF, Befroy D, Dufour S, Dziura J, Ariyan C, Rothman DL et al Mitochondrial dysfunction in the elderly: possible role in insulin resistance. Science. 2003;300(5622):1140–2. 10.1126/science.1082889 12750520 PMC3004429

[24] Mootha VK, Lindgren CM, Eriksson KF, Subramanian A, Sihag S, Lehar J et al PGC-1alpha-responsive genes involved in oxidative phosphorylation are coordinately downregulated in human diabetes. Nature genetics. 2003;34(3):267–73. 10.1038/ng1180 12808457

[25] Patti ME, Butte AJ, Crunkhorn S, Cusi K, Berria R, Kashyap S et al Coordinated reduction of genes of oxidative metabolism in humans with insulin resistance and diabetes: Potential role of PGC1 and NRF1. Proceedings of the National Academy of Sciences of the United States of America. 2003;100(14):8466–71. 10.1073/pnas.1032913100 12832613 PMC166252

[26] Stuart CA, McCurry MP, Marino A, South MA, Howell ME, Layne AS et al Slow-twitch fiber proportion in skeletal muscle correlates with insulin responsiveness. The Journal of clinical endocrinology and metabolism. 2013;98(5):2027–36. 10.1210/jc.2012-3876 23515448 PMC3644602

[27] Schweitzer GG, Castorena CM, Hamada T, Funai K, Arias EB, Cartee GD. The B2 receptor of bradykinin is not essential for the post-exercise increase in glucose uptake by insulin-stimulated mouse skeletal muscle. Physiol Res. 2011;60(3):511–9. 21401298 10.33549/physiolres.932085 PMC3535296

[28] Kim KH, Jeong YT, Oh H, Kim SH, Cho JM, Kim YN et al Autophagy deficiency leads to protection from obesity and insulin resistance by inducing Fgf21 as a mitokine. Nature medicine. 2013;19(1):83–92. 10.1038/nm.3014 23202295

[29] Holloszy JO, Booth FW. Biochemical adaptations to endurance exercise in muscle. Annual review of physiology. 1976;38:273–91. 10.1146/annurev.ph.38.030176.001421 130825

[30] Ojuka EO. Role of calcium and AMP kinase in the regulation of mitochondrial biogenesis and GLUT4 levels in muscle. The Proceedings of the Nutrition Society. 2004;63(2):275–8. 10.1079/PNS2004339 15294043

[31] Wu Z, Puigserver P, Andersson U, Zhang C, Adelmant G, Mootha V et al Mechanisms controlling mitochondrial biogenesis and respiration through the thermogenic coactivator PGC-1. Cell. 1999;98(1):115–24. 10.1016/S0092-8674(00)80611-X 10412986

[32] Contarteze RV, Manchado Fde B, Gobatto CA, De Mello MA. Stress biomarkers in rats submitted to swimming and treadmill running exercises. Comparative biochemistry and physiology Part A, Molecular & integrative physiology. 2008;151(3):415–22. 10.1016/j.cbpa.2007.03.005 17428717

[33] Fischer M, Lieb W, Marold D, Berthold M, Baessler A, Lowel H et al Lack of association of a 9 bp insertion/deletion polymorphism within the bradykinin 2 receptor gene with myocardial infarction. Clinical science. 2004;107(5):505–11. 10.1042/CS20040129 15301669

[34] Braun A, Kammerer S, Maier E, Bohme E, Roscher AA. Polymorphisms in the gene for the human B2-bradykinin receptor. New tools in assessing a genetic risk for bradykinin-associated diseases. Immunopharmacology. 1996;33(1–3):32–5. 8856111 10.1016/0162-3109(96)00079-3

[35] Popadic Gacesa JZ, Momcilovic M, Veselinovic I, Brodie DA, Grujic NG. Bradykinin type 2 receptor -9/-9 genotype is associated with triceps brachii muscle hypertrophy following strength training in young healthy men. BMC musculoskeletal disorders. 2012;13:217 10.1186/1471-2474-13-217 23127247 PMC3531309

